# The Role of 3′ to 5′ Reverse RNA Polymerization in tRNA Fidelity and Repair

**DOI:** 10.3390/genes10030250

**Published:** 2019-03-26

**Authors:** Allan W. Chen, Malithi I. Jayasinghe, Christina Z. Chung, Bhalchandra S. Rao, Rosan Kenana, Ilka U. Heinemann, Jane E. Jackman

**Affiliations:** 1 Department of Biochemistry, The University of Western Ontario, 1151 Richmond Street, London, ON N6A 5C1, Canada; achen325@uwo.ca (A.W.C.); cchung88@uwo.ca (C.Z.C.); rkenana@uwo.ca (R.K.); 2 Department of Chemistry and Biochemistry, Center for RNA Biology, Ohio State Biochemistry Program, The Ohio State University, Columbus, OH 43210, USA; jayasinghearachchige.1@osu.edu (M.I.J.); brao@arrakistx.com (B.S.R.)

**Keywords:** reverse polymerization, tRNA editing, tRNA repair, protein engineering, synthetic biology

## Abstract

The tRNA^His^ guanylyltransferase (Thg1) superfamily includes enzymes that are found in all three domains of life that all share the common ability to catalyze the 3′ to 5′ synthesis of nucleic acids. This catalytic activity, which is the reverse of all other known DNA and RNA polymerases, makes this enzyme family a subject of biological and mechanistic interest. Previous biochemical, structural, and genetic investigations of multiple members of this family have revealed that Thg1 enzymes use the 3′ to 5′ chemistry for multiple reactions in biology. Here, we describe the current state of knowledge regarding the catalytic features and biological functions that have been so far associated with Thg1 and its homologs. Progress toward the exciting possibility of utilizing this unusual protein activity for applications in biotechnology is also discussed.

## 1. Introduction

**Figure 1:**
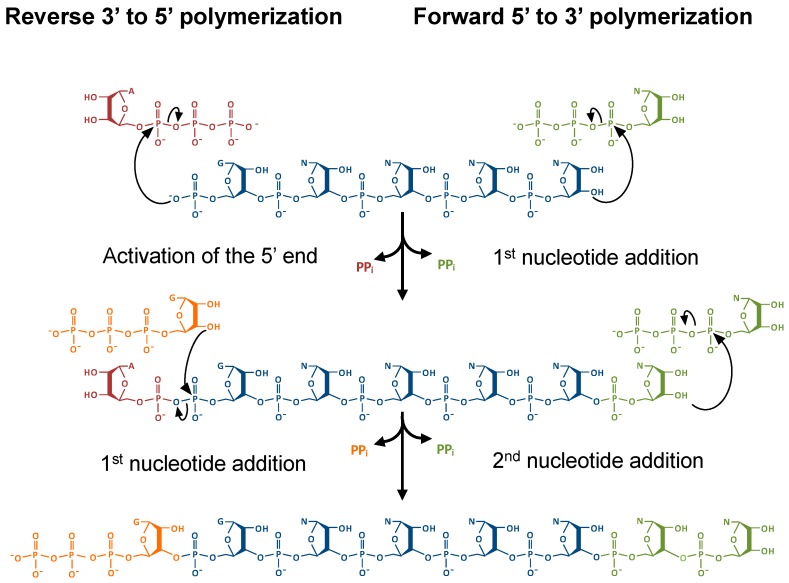
Forward 5′ to 3′ and reverse 3′ to 5′ polymerization are mechanistically similar. Ligation of a nucleotide to an RNA strand utilizes chemical energy that has been stored in the high-energy phosphoanhydride bonds in the nucleotide triphosphate. During forward polymerization, the triphosphate of the incoming nucleotide is hydrolyzed and provides energy to form a phosphodiester bond. For reverse polymerization, an initial activation step of a monophosphorylated RNA 5′ end (such as by adenylylation, which is shown here) is required; subsequent nucleotide additions utilize energy derived from hydrolysis of the phosphodiester bond of the RNA 5′-end triphosphate.

During DNA strand synthesis, 5′ to 3′ polymerization conveys significant advantages, particularly in the removal of mismatched nucleotides by the exonuclease activity of DNA polymerases [1,2]. This 3′ to 5′ nucleotide removal regenerates a polymerizable 3′-OH end, whereas the removal of a 5’ nucleotide generates a 5’-monophosphate, which is thought to be incapable of immediate replacement with another nucleotide (Figure 1) [2]. However, 3′ to 5′ or reverse polymerization is possible, despite the restrictions of extending a 5’-monophosphate end [3]. For such a 3′-5′ polymerase to function, the 5′-monophosphate end could be activated by ATP, creating an intermediate that is competent for nucleophilic attack by the 3′-OH of an incoming nucleotide, which is then a mechanistically similar process to forward polymerization (Figure 1) [4,5]. Indeed, enzymes that are capable of catalyzing this reverse polymerase reaction, which are members of the tRNA^His^ guanylyltransferase (Thg1) superfamily, have been recently discovered [6,7,8,9,10]. Despite the possible advantages of reverse polymerization, such as coupled leading strands in DNA replication as opposed to the generation of Okazaki fragments, 3′ to 5′ polymerization appeared to be more limited in biology, and was initially only observed to function in several reactions associated with tRNA repair and processing. However, newer data suggests broader substrate specificity exhibited by some members of the Thg1 family known as Thg1-like proteins (TLPs), thus raising the potential for additional roles for 3′ to 5′ polymerization yet to be discovered. 

## 2. Thg1 Maintains tRNA^His^ Aminoacylation Fidelity

### 2.1. G_-1_ is a tRNA^His^ Identity Element

Translational accuracy is dependent upon both tRNA selection by the ribosome and prior tRNA recognition and charging by a specific cognate aminoacyl-tRNA synthetase (aaRS) [11,12]. The side chain of the histidine residue, with its associated acid–base properties that contribute to both the protein structure and catalytic mechanisms, must be reliably and appropriately incorporated during translation [11,13]. Therefore, the aminoacylation of an amino acid to its respective tRNA must be specific, especially in the case of tRNA^His^ due to its particular properties that are difficult to chemically mimic with another natural amino acid, possibly explaining why tRNA^His^ relies on several key identity elements for proper aminoacylation.

**Figure 2:**
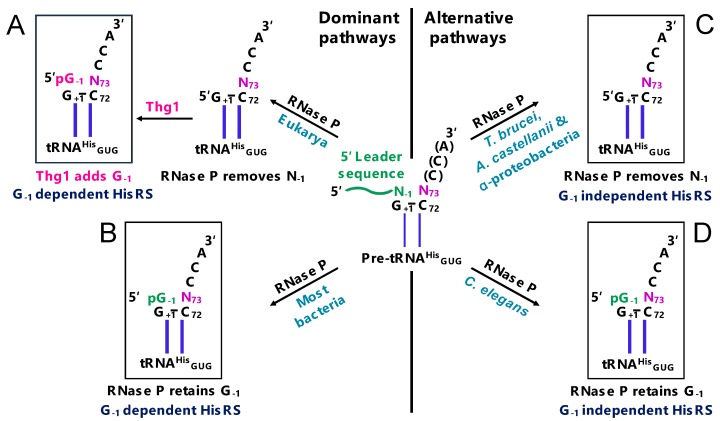
Different pathways to establish tRNA^His^ identity. (**A**) tRNA^His^ identity in many eukaryotes. RNase P removes N_−1_ from pre-tRNA^His^ during the removal of 5′ leader sequence (shown in green). Then, tRNA^His^ guanylyltransferase (Thg1) post-transcriptionally adds G_−1_ (shown in pink). Histidyl-tRNA synthetase (HisRS) recognizes the Thg1-incorporated G_-1_ for the accurate histidylation of tRNA^His^ in eukaryotes. (**B**) tRNA^His^ identity in bacteria. G_−1_ is encoded in tRNA^His^ genes in most bacteria, and RNase P retains the genomically encoded G_−1_ during the removal of the 5′ leader sequence. HisRS recognizes the RNase P-retained G_-1_ for the accurate histidylation of tRNA^His^ in bacteria. (**C**) tRNA^His^ identity in several groups of α-proteobacteria, *Trypanosoma brucei* and *Acanthamoeba castellanii*. RNase P removes N_−1_ from precursor tRNA^His^ during the removal of the 5′ leader sequence. An atypical HisRS in these organisms is capable of aminoacylating G_−1_-lacking tRNA^His^ during histidylation. (**D**) tRNA^His^ identity in *Caenorhabditis elegans* (an atypical eukaryote). G_−1_ is encoded in tRNA^His^ genes, and RNase P retains the genomically encoded G_−1_, which is similar to bacteria. HisRS in *C. elegans* is capable of aminoacylating both G_−1_-containing and G_−1_-lacking tRNA^His^.

For tRNA^His^ in all domains of life, one of these identity elements is a distinctive extra guanine nucleotide at the 5′-end (the –1 position) [14] (Figure 2). The 5′ guanylate residue at the –1 position (G_−1_) serves as a key identity element for histidyl-tRNA synthetase (HisRS), which charges tRNA^His^ with its respective histidine [15,16,17]. In bacteria and many archaea, the G_−1_ residue is genomically encoded and transcribed in the precursor tRNA transcript, and subsequent cleavage of the 5′ leader sequence by ribonuclease P (RNase P) yields a mature tRNA^His^ with its identity-establishing G_-1_ element (tRNA^His^G_−1_) (Figure 2) [14,18,19].

In many eukaryotes, the establishment of tRNA^His^ identity follows a different pathway. The G_−1_ residue is not encoded in the tRNA^His^ gene, but rather is added post-transcriptionally by Thg1 following RNase P cleavage (Figure 2A) [20,21]. Consistent with the necessity for the G_−1_ determinant of tRNA^His^ identity in protein fidelity, deletion or silencing of the gene encoding Thg1 results in severe growth defects in yeast, humans, and *Dictyostelium discoideum*, which is consistent with the wide conservation of Thg1 in Eukarya [21,22,23]. The addition of G_−1_ by Thg1 in eukaryotes occurs opposite of the conserved terminal A_73_ and is the result of a non-templated 3′-5′ addition [20,21]. A few exceptions to the necessity of G_−1_ have already been identified; a group of α-proteobacteria and several protozoan eukaryotes (*Acanthamoeba castellanii* and *Trypanosoma brucei)* do not genomically encode or post-transcriptionally add the G_−1_ identity element; the absence of the otherwise highly conserved extra nucleotide is accommodated by an atypical HisRS recognition of tRNA^His^ (Figure 2, right panel) [24,25,26,27].

### 2.2. Thg1 Structurally Resembles Canonical 5′ to 3′ Polymerases

**Figure 3:**
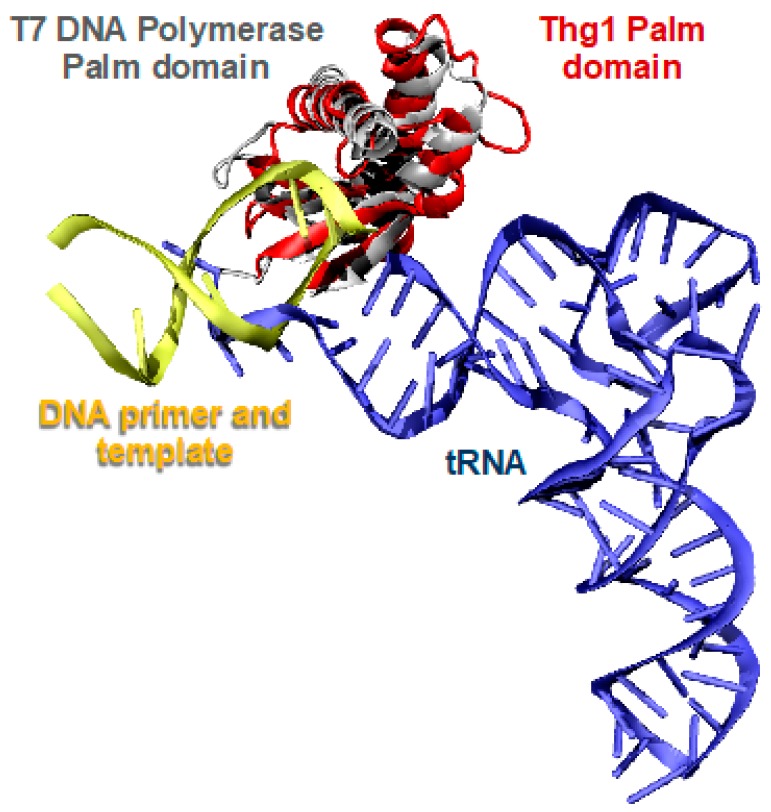
Forward and reverse polymerization is catalyzed by structurally similar enzymes but require opposing substrate orientation. The tRNA substrate (blue) of Thg1 (red) is in an opposing orientation compared to the DNA substrate and template (yellow) approaching the T7 DNA polymerase palm domain (grey).

Human Thg1 (HsThg1) is encoded by 269 amino acids with a calculated molecular weight of 32 kDa, but purification by gel exclusion chromatography eluted a protein of a molecular weight of ~165 kDa. This suggests the formation of a higher order multimer in solution, and is consistent with existing determinations of dimer-of-dimer tetrameric forms of active Thg1 enzymes [4,5,28,29,30]. Despite Thg1 enzymes sharing no obvious sequence similarity to known polymerases, a surprising structural homology exists between Thg1 and T7 DNA polymerase and DNA polymerase II (family pol I and pol α, respectively), as the enzymes share the same catalytic palm domain (Figure 3) [4,5]. The superposition of Thg1 with the aforementioned polymerases displays similar positioning of the three conserved carboxylate residues in the polymerase active site to the pol I family; HsThg1 carboxylates D29, D76, and E77 correspond to T7 DNA polymerases D475, D654, and E655. This similar carboxylate positioning suggests that the Thg1 mechanism of reverse polymerization may share features with the forward polymerization of the pol I family. Moreover, in the context of the overall structure, each monomeric subunit resembles a polymerase “hand” shape with the palm and fingers domains. This discovery revealed that forward and reverse polymerization can be accommodated by the same catalytic palm domain [4,5].

Crystal structures of eukaryotic Thg1 enzymes showed that monomeric Thg1 is composed of a six-strand antiparallel β-sheet flanked by three or four α-helices on each side, along with a protruding long arm composed of two antiparallel β-strands (Figure 3). Each monomer forms a dimer, mediated by salt bridge formation and hydrogen bonding between the β-sheet and an α-helix [4,5]. Alanine scanning mutagenesis in *Saccharomyces cerevisiae* Thg1 (ScThg1) of conserved residues responsible for mediating dimer formation has shown strongly diminished G_−1_ addition activity [4,31]. Dimer-of-dimer formation stems from initial dimer formation; the α-helix of the N-terminus in monomer A interacts with the nucleotide binding site of monomer B and ultimately forms an intertwined N-terminus segment, which again interacts with a symmetrical dimer to form its active tetrameric form [4,5]. The crystal structures of Thg1 from *Candida albicans* and *S. cerevisiae* corroborate similar findings in protein folding and quaternary structure, which is suggestive that Thg1 variants fold similarly, and are active in tetrameric form [4,5,28,30]

The cocrystal structure of *C. albicans* Thg1 (CaThg1) in complex with tRNA subsequently provided insight into the mechanistic details of reverse polymerization. Compared to forward polymerases, such as the T7 DNA polymerase, the RNA substrate of Thg1 approaches the catalytic palm domain from the opposing site (Figure 3) [5]. Thus, the direction of polymerization is dependent on the direction of substrate approach to the catalytic core; Thg1 forces incoming nucleotides to approach from the opposite orientation of canonical forward polymerases, which is reflective of the overall domain organization of the enzyme (Figure 3). The fingers domain, which in part forms the nucleotide-binding site, is situated on opposite sides of the palm domain in Thg1, forcing a reversed substrate approach. Thus, the domain organization of Thg1 cannot accommodate forward polymerization, just as forward polymerases do not accommodate reverse polymerization [5]. Overall, these data revealed the molecular basis of reverse nucleotide addition, and showed that while reverse and forward polymerization can be catalyzed by the same catalytic core, substrate orientation may be the deciding factor in determining the directionality of nucleotide addition.

### 2.3. The Molecular Basis for tRNA Recognition

Thg1 recognizes tRNA^His^ through its GUG anticodon, as demonstrated by the ability of Thg1 to add G_−1_ to a mutagenized tRNA^Phe^ that has been altered to contain the His anticodon (tRNA^Phe^_GUG_) and subsequently validated through a cocrystal structure of *C. albicans* Thg1 in complex with tRNA^Phe^_GUG_. The coordination of tRNA molecules occurs in a molar ratio of 4:2, where two tRNA molecules bind a Thg1 tetramer in parallel orientation, and each tRNA is coordinated by three subunits of the tetramer [5]. Common identity elements of the coordinated tRNA interact with the Thg1 tetramer; the acceptor stem is coordinated by the intertwined N-terminus of a Thg1 dimer, and the T arm is situated near polar residues on the rear surface of the catalytic core of the same dimer. This surface interaction in tRNA stabilization is analogous to the thumb domain in canonical forward polymerases. However, the anticodon, which is another distinctive identity element, is coordinated by the opposite dimer, and the G_−1_ addition of this tRNA occurs solely in one dimer of the tetramer, specifically, the dimer that coordinates the acceptor stem and T arm positioning. The structural superposition of a crystal structure of ScThg1 and CaThg1-tRNA^Phe^_GUG_ discovered a conserved secondary structure in the fingers domain, and suggested conserved dual RNA-binding surfaces that were originally elucidated in CaThg1. Both the acceptor stem’s sugar phosphate backbone and the anticodon loop are coordinated by the fingers domain; the latter fingers–anticodon interaction is base-specific [5].

Recognition of the tRNA^His^ anticodon has been rationalized structurally through the CaThg1:tRNA^Phe^_GUG_ complex [5]. The G_34_, U_35_, and G_36_ nucleotides that make up the tRNA^His^ anticodon were all observed to be coordinated by specific residues of CaThg1. All three anticodon bases are tightly coordinated, and mutations of the coordinating amino acids lead to a disruption or reduction in enzyme activity [4,5,31]. The anticodon loop structure itself is stabilized by interactions with the sugar phosphate backbone of U_35_. Anticodon base G_36_ is coordinated in a groove formed by two α-helices flanking the central β-sheet and stabilized by a stacking interaction with H154, which is an essential residue in the eukaryotic-specific sequence motif HINNLYN [5,32]. The structure of Thg1 in complex with tRNA^Phe^_GUG_ suggests the molecular basis for the fairly stringent dependence of Thg1 on anticodon recognition, which is in agreement with its biological function of establishing tRNA^His^ identity.

### 2.4. The Molecular Basis for Non-Watson–Crick G_−1_ Addition: tRNA Activation

**Figure 4:**
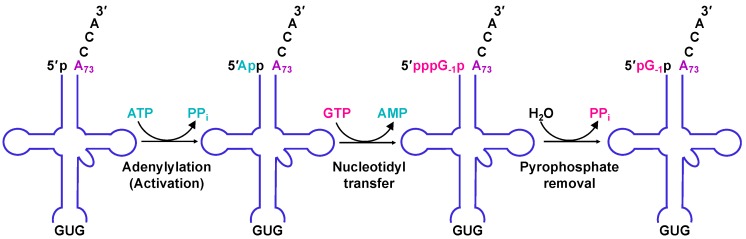
Eukaryotic Thg1 catalyzes a three-step reaction. ScThg1 adds G_-1_ in three consecutive steps. First, the 5′-end of a monophosphorylated tRNA^His^ (p-tRNA^His^) is activated by an ATP to generate a 5′-adenylylated tRNA^His^ (App–tRNA^His^) intermediate (adenylation activation). Second, the 3′-hydroxyl of an incoming GTP nucleotide attacks the 5′-end of the App–tRNA^His^, which then releases AMP and adds the GTP to yield a triphosphorylated tRNA^His^ (pppG_–1_p–tRNA^His^) intermediate (nucleotidyl transfer). Third, pyrophosphate (PP_i_) from the pppG_−1_p–tRNA^His^ is released, creating a mature pG_−1_p–tRNA^His^ (pyrophosphate removal).

The resemblance between canonical forward polymerase structures and Thg1 in the overall structure raises questions about how exactly Thg1 performs reverse polymerization. In forward polymerization, the catalytic core coordinates the two catalytic metal ions with the first Mg^2+^ (Mg^2+^ A) promoting deprotonation of the 3′-OH in the polymerizing DNA strand, and the second Mg^2+^ (Mg^2+^ B) coordinating the triphosphate moiety of the incoming nucleotide. By analogy, Mg^2+^ A and Mg^2+^ B in Thg1 family polymerases use similar chemical features to catalyze the first step of G_-1_ addition by activating the 5′-end of tRNA^His^ with ATP (Figure 1 and Figure 4). In this case, Mg^2+^ A would promote the nucleophilic attack of the tRNA 5′-phosphate on the α-phosphate of the activating ATP nucleotide, which is also bound by Mg^2+^ B. The exact mechanism of the activation step is not entirely known, but the GTP-bound crystal structures of HsThg1, ScThg1, and CaThg1 have elucidated binding interactions in the activation of the nucleotide-binding site and revealed a possible mechanism of how Thg1 differentiates between ATP and GTP in the initial activation of tRNA^His^ [4,5]. Nucleotidyl transfer in HsThg1 and ScThg1 is mediated largely by guanine base stacking against A37 and F42. The interaction is furthered by hydrogen bonding via amide, carbonyl, and the side chain moieties of D47, A43, and H34, respectively [4,5]. The superimposition of CaThg1–ATP and CaThg1–GTP cocrystal structures revealed that the nucleotide responsible for initial activation, ATP, resides deeper in the nucleotide-binding pocket than GTP, and interacts directly with D47 and K44. Mutational analysis of a ScThg1 D44A mutant greatly decreased the catalytic efficiency, suggesting that the D44 interaction is involved in G_−1_ addition activation [33].

### 2.5. The Molecular Basis for Non-Watson–Crick G_−1_ Addition: Nucleotidyl Transfer

Thg1 family enzymes have a distinct site (at least partially separable from the activation site) for the binding of the incoming nucleotide used for the nucleotidyl transfer step (Figure 1 and Figure 4). This can be clearly seen in HsThg1–dGTP and CaThg1–GTP crystal structures [4,5]. This observation is further supported by the kinetic data showing distinct functions for highly conserved residues in ScThg1. ScThg1 residues R27, K96, and R133 play a more significant role during the nucleotidyl transfer step based on more significant defects in the observed rate of this reaction with alanine variants compared to the other steps in G_−1_ addition [34]. However, these three residues only interact with the triphosphate moiety of the partially visualized incoming dGTP nucleotide in the HsThg1–dGTP crystal structure and the completely visualized GTP in the CaThg1–GTP structure. Due to the lack of direct contacts with the base or ribose of the GTP bound to the nucleotidyl transfer site in the CaThg1–GTP structure, neither of these structures explain how Thg1 specifically positions or recognizes its highly preferred nucleotide, GTP, over any other NTP, as observed repeatedly in many in vitro assays. Thus, the precise mechanism of Thg1-catalyzed nucleotidyl transfer to create the non-Watson–Crick G_−1_–A_73_ base pair step is yet to be determined. 

### 2.6. The Molecular Basis for Non-Watson–Crick G_-1_ Addition: Pyrophosphate Removal

In the final step of the reaction catalyzed during tRNA^His^ maturation by Thg1, the enzyme removes the 5′-pyrophosphate from the added G_−1_ nucleotide, yielding the 5′-monophosphate that is required for recognition by HisRS (Figure 4) [17,21,33]. This reaction requires the same two metal ion active sites as used for the previous two steps of the reaction, since alanine mutations to the metal coordinating carboxylate residues completely eliminate the ability of the enzyme to catalyze this reaction in vitro [33]. However, additional residues that participate directly in the chemistry of this reaction have not yet been identified [33,35]. Interestingly, the removal of this pyrophosphate also removes an activated 5′-triphosphorylated end that could be extended further by the enzyme in the reverse polymerase reaction. Consistent with this idea, removal of the 5′-pyrophosphate by ScThg1 occurs much more efficiently in the context of the non-Watson–Crick base paired 5′-ends and 3′-ends of the tRNA than with a Watson–Crick base-paired end, thus preventing the further extension of this tRNA and limiting addition to the single essential G_−1_ nucleotide [36].

### 2.7. Maintenance of tRNA^His^ Fidelity

Maintaining tRNA identity is essential for translational fidelity, although a certain amount of mistranslation can be tolerated by the cell [12,37]. In eukaryotes, G_−1_ addition is essential to establish tRNA^His^ identity, and consequently, Thg1 is required for cell survival. In *S. cerevisiae*, the conditional depletion of Thg1 leads to the accumulation of unguanylated and uncharged tRNA^His^ and growth arrest [8]. Interestingly, tRNA^His^ in Thg1-depleted cells contains elevated levels of a 5-methylcytidine (m^5^C) modification at residues C_48_ and C_50_ [38]. While the change in nucleoside methylation is specific to tRNA^His^, it is most likely the result of the growth arrest rather than a direct consequence of G_−1_ depletion. Upon conditional Thg1 depletion, these cells are arrested in prophase or G2. Thg1 was also shown to interact with the Orc2 component of the origin recognition complex, and loss of this interaction impairs DNA replication and nuclear division, although the precise molecular basis for this apparent connection of Thg1 to DNA metabolism remains unknown [39]. Thus, not surprisingly, Thg1 activity is essential in eukaryotes such as *S. cerevisiae*, which depend on Thg1 to maintain tRNA^His^ identity.

## 3. Thg1-Like Proteins Function in tRNA Repair

### 3.1. Thg1-Like Proteins Are Functionally and Phylogenetically Distinct from Thg1

The biological function of Thg1 has been investigated in detail, but Thg1-like proteins (TLPs, which are also referred to as archaeal-type Thg1) only more recently gained attention. First identified in archaea, homologs have now been identified and characterized from several bacteria and eukaryotes. While related in sequence and structure, Thg1 and TLPs are phylogenetically and functionally distinct [32,40]. Where Thg1 is essential in many eukaryotes to establish tRNA^His^ identity through the post-transcriptional addition of G_−1_, most bacteria and most archaea instead genomically encode the G_−1_ identity element (Figure 2) [14,18,19,20,41]. Interestingly, in vitro analysis of bacterial and archaeal Thg1 homologs demonstrated that they are capable of catalyzing an analogous addition of G_−1_ to bacterial/archaeal tRNA^His^ transcripts that lack the G_−1_ nucleotide, which would necessarily require the templated polymerization of G_−1_ opposite to the C_73_ that is universally found in these tRNA^His^ [23,40]. Using these different biochemical capabilities and distinct sequence features as a functional classification system, Thg1 superfamily enzymes can thus be classified into two groups. The first group entails the bona fide Thg1 homologs, which are found only in Eukarya, and post-transcriptionally establishes tRNA^His^ identity via G_−1_. The second group includes TLP homologs that are found in all three domains of life, and have not yet been implicated biologically in tRNA^His^ identity, but for which this function would be in many cases redundant, if at all observed in vivo [23,32,40,42,43,44].

A phylogenetic analysis of candidate Thg1/TLP genes from all domains of life have grouped nearly all eukaryotic Thg1 variants into a single group; a second clade containing eukaryotic TLPs also contains archaeal and bacterial TLPs, which further supports the classification of Thg1 and TLPs as distinct enzymes [32,43,45]. A secondary trend further groups bacterial and archaeal Thg1 into two separate phylogenetic groups, and suggests that bacterial Thg1 was not descended from earlier bacterial ancestors [32,43,45].

### 3.2. The Discovery of TLPs in Bacteria and Archaea

The first characterization of a non-eukaryotic Thg1 homolog was carried out in *Methanosarcina acetivorans* [43]. Initially, the enzyme was annotated as two open reading frames split by a UAG Stop codon. While the enzyme activity of the split enzyme could be reconstituted in vitro, the protein is translated into a single protein in vivo, linking the two frames by a genetically encoded pyrrolysine at the UAG codon [43]. While in *M. acetivorans* the UAG codon could still be signaling for translation termination, it is unlikely that Thg1 is translated as two halves in vivo [46]. To date, Thg1 homologs belonging to the TLP clade have now been characterized biochemically from a diverse range of eight different bacterial and archaeal species. The common ability of all enzymes to catalyze strictly templated 3′ to 5′ addition reactions has been universally observed in assays of these enzymes with both tRNA^His^ and non-tRNA^His^ substrates [23,29,35,40,45,47]. The question of the precise nature of the biological substrates of these enzymes in their relevant organisms has not been entirely addressed, but some likely possibilities based on in vitro characterization so far include analogous tRNA^His^ maturation reactions to those employed by eukaryotic Thg1 (albeit in many cases redundant with co-transcriptional mechanisms of obtaining the G_−1_ nucleotide) and/or alternative RNA repair reactions catalyzed on substrates such as 5′-truncated tRNAs that remain to be identified. 

The idea that bacterial and archaeal homologs of Thg1 might participate in G_−1_ addition to tRNA^His^ was an obvious extension of the known function of these enzymes in eukaryotes, and the initial in vitro characterization of several enzymes demonstrated activities that were consistent with this type of role [40,45,47]. It is notable that endogenous tRNA^His^ genes in these species universally encoding a C_73_ nucleotide in place of the A_73_ found in eukaryotes was also consistent with the known preference of these enzymes to catalyze Watson–Crick-dependent 3′ to 5′ addition reactions. Thus, it was not surprising to see that TLPs were not able to efficiently add G_-1_ to wild-type A_73_-containing eukaryotic tRNA^His^ substrates, either in vitro or in vivo [23,40,45,47]. Interestingly, the *Bacillus thuringiensis* TLP homolog (BtTLP) is unusual compared to several other tested enzymes of this family, in that it is able to support the growth of a yeast *Δthg1* strain, presumably by acting in place of ScThg1 on tRNA^His^ [23,40,47]. Indeed, sequences of tRNA^His^ derived from the BtTLP-complemented *S. cerevisiae Δthg1* strain reveal that the tRNA contains almost exclusively U_−1_, instead of the canonical G_−1_ residue [48]. In this case, the BtTLP evidently maintains its preference for incorporating Watson–Crick base-paired nucleotides by incorporating U_−1_ across from the A_73_ discriminator nucleotide. Although the minor growth defect of the BtTLP-expressing strains may then be attributed to the presence of the non-canonical U_−1_ nucleotide on tRNA^His^, the ability of *S. cerevisiae* HisRS to accept other N_−1_-containing tRNA^His^ substrates in vitro with only a fivefold to sixfold loss in efficiency is consistent with the observed viability of the BtTLP-expressing strain. 

### 3.3. TLPs Catalyze Multiple Nucleotide Additions

Interestingly, TLP homologs differ in terms of the number of nucleotide additions that are observed in vitro with tRNA transcripts mimicking the mature tRNA^His^ that could be a substrate for this kind of activity in vivo. The ability to add multiple 5′-nucleotides to these types of tRNA^His^ is a direct consequence of the presence of a C_73_ discriminator, which results in the formation of three consecutive C-nucleotides in the 3′-C_73_CCA end. These types of substrates were initially shown to cause multiple G-addition reactions even in the context of eukaryotic Thg1, and several bacterial/archaeal homologs (the TLPs from *Methanopyrus kandleri*, *Pyrobaculum aerophilum,* and *B. thuringiensis*) behave similarly, catalyzing multiple G-additions [9,23,35,40,47]. However, other homologs (such as from *Myxococcus xanthus*, *Methanothermobacter thermoautotrophicus*, *Methanosarcina barkeri*, and *M. acetivorans*) appear to limit nucleotide addition to only a single guanine addition, despite the presence of an extended C-template at the tRNA 3′-end [40]. The molecular basis for these distinct behaviors is not yet known, but suggests that not all homologs in Bacteria and Archaea would react similarly in terms of any possible role in the maturation or maintenance of tRNA^His^ [9,23,35,40,47].

The observation of alternative RNA substrate specificity exhibited by many enzymes in the TLP clade also suggested the possibility of non-tRNA^His^ related functions for these enzymes. The identification of certain biochemical properties that distinguish TLPs from bona fide Thg1 counterparts lent support to these ideas. In addition to the previously described ability of TLPs to efficiently catalyze Watson–Crick base pair-dependent addition reactions, all of the bacterial and archaeal homologs investigated to date exhibit the preferential repair of 5′-truncated tRNAs instead of 5′ nucleotide addition to full-length tRNA^His^ [23,29,35]. This tRNA repair activity is not species-specific, as opposed to G_-1_ addition in tRNA^His^ identity establishment by bona fide Thg1 [23,26,31,42]. *M. acetivorans* TLP (MaTLP) has also demonstrated similar 5′-truncated tRNA repair as previously described with BtTLP; with preference to additions forming Watson–Crick base pairs [23,29,40]. Similarly, *Ignicoccus hospitalis* catalyzes an extended tRNA repair on truncated RNA substrates in vitro, adding up to 13 nucleotides in a templated reaction to restore a full tRNA^His^ [35]. These data indicate that TLP function may indeed be found in tRNA repair rather than G_−1_ addition.

### 3.4. TLPs in Eukaryotes: Multiple TLPs Encoded by Dictyostelium Discoideum

**Figure 5:**
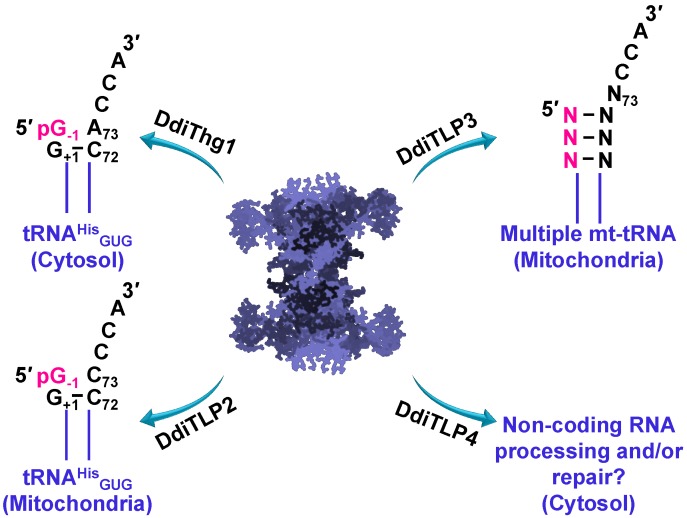
Non-redundant physiological roles for Thg1 and Thg1-like proteins (TLPs) in *Dictyostelium discoideum* tRNA 5′-editing. DdiThg1 catalyzes cytosolic tRNA^His^ maturation by incorporating G_−1_ across from A_73_. DdiTLP2 catalyzes mitochondrial tRNA^His^ maturation by incorporating G_−1_ across from C_73_. DdiTLP3 catalyzes mitochondrial tRNA 5′-editing by repairing 5′-truncated tRNAs resulting from the removal of one or more mismatched nucleotides encoded in the precursor tRNAs. DdiTLP4 function remains unknown to date, but potentially this essential cytosolic enzyme is involved in non-coding RNA processing and/or repair in *D. discoideum*.

Analysis of the eukaryotic slime mold, *Dictyostelium discoideum*, revealed that at least four Thg1 family enzymes appear to be present in the proteome (Figure 5). One of these genes is a bona fide Thg1 (DdiThg1), catalyzing the G_−1_ addition to tRNA^His^, and thus establishing tRNA^His^ identity [42,44]. The remaining three genes are characterized by sequence similarity and phylogeny as TLPs. Two of these TLPs (DdiTLP2 and DdiTLP3) are mitochondrial enzymes that catalyze distinct and non-redundant functions to add G_−1_ to mitochondrial tRNA^His^ (DdiTLP2) or to repair the 5′-end of mitochondrial tRNA (mt-tRNA) during a process known as tRNA 5′-editing (DdiTLP3) [44]. Both of these reactions take advantage of the 3′ to 5′ polymerase function that is the biochemically-preferred activity of TLP enzymes. 

Although DdiTLP2 catalyzes a reaction that is on the surface very similar to that of a bona fide Thg1, there are several critical features that distinguish these two activities, which is consistent with the evolutionary distinct nature of these two enzymes. First, DdiTLP2 does not use the tRNA^His^ GUG anticodon to recognize the tRNA for the addition of the G_−1_ nucleotide, and its specific mechanism of tRNA^His^ recognition remains unknown [44]. Second, the reaction catalyzed by DdiTLP2 is not essential for specifying tRNA^His^ identity, since *D. discoideum Δdditlp2* deletion strains are viable, but completely lack the G_−1_ nucleotide on the mt-tRNA^His^ [44]. DdiTLP3’s role in tRNA 5′-editing also utilizes 3′ to 5′ polymerase function, but to repair mt-tRNA that have been truncated at their 5′-ends due to the removal of one or more incorrectly base-paired nucleotides encoded in the precursor tRNA [49]. This 5′-end repair step is essential for *D. discoideum*, and likely for many other single-celled eukaryotes that similarly encode mt-tRNA with 5′-mismatches [34,47,49,50,51,52,53,54]. Presumably, the TLPs encoded by these species are capable of participating in 5′-editing, although the identity of specific enzymes that participate in this process has so far only been demonstrated in *D. discoideum* [32,33,41,42,43,44]. Interestingly, the third TLP encoded in *D. discoideum* (DdiTLP4) catalyzes an essential function that remains unknown (Figure 5), although its ability to catalyze 3′ to 5′ polymerase activity on non-tRNA RNA substrates broadens the potential impact of these enzymes in terms of RNA processing and/or repair [44]. The continued functional characterization of TLP enzymes from diverse domains of life will be needed to provide a comprehensive picture of all of the biological reactions associated with this unusual family of enzymes (Figure 5).

### 3.5. Structural Comparison of Thg1 and TLPs

**Figure 6:**
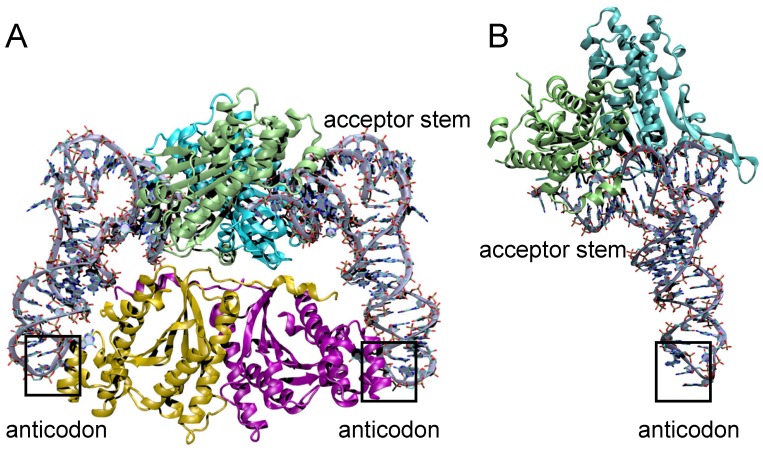
Structural comparison of Thg1 and TLPs. (**A**) The tetrameric eukaryotic Thg1 (monomers in green, teal, yellow and pink) coordinates two tRNAs molecules (grey) and binds both to the anticodon and the acceptor stem. (**B**) *Methanosarcina acetivorans* TLP forms a dimer (monomers in green and teal) coordinating on tRNA (grey). While the dimer binds the acceptor stem and T arm of its tRNA substrate, the anticodon loop is not coordinated by TLP enzymes.

The crystal structures of several members of the TLP clades of the Thg1 superfamily have been solved, and suggest many commonalities with Thg1, including an overall structural similarity to the *C. albicans* and *Homo sapiens* enzymes and the persistence of dimer-of-dimer quaternary structures [28,29]. MaTLP assembles as a dimer-of-dimers similar to bona fide Thg1, yet uses a different mechanism of tRNA coordination. CaThg1 coordinates tRNA between both dimers of the tetramer, and binds Thg1–tRNA via 4:2 stoichiometry [54]. MaTLP independently binds one tRNA molecule per dimer, and does not seem to coordinate anticodon recognition by the opposing dimer, which is consistent with the notion of TLP repair activity (Figure 6) [23,40]. The present structures of the MaTLP and bona fide Thg1 variants differ most in tRNA coordination; the anticodon loop is not coordinated by the opposing dimer’s fingers domain in TLPs [5,29]. MaTLP binds only the acceptor stem and T arm of its tRNA substrate; the flexible β-hairpin that coordinates the T arm has been speculated to enable the recognition of tRNA substrates with truncated acceptor stems [29]. The tRNA acceptor stem and T arm are coordinated by separate monomers in the dimer; the acceptor stem was hydrogen bonded to R136 and D137, and the triphosphate moiety at the 5′-end of the tRNA was bonded to the D21–K26 region and additionally coordinated by two Mg^2+^, which is similar to tRNA coordination by CaThg1 [5,29]. Expectedly, divalent cations of the MaTLP active site are coordinated by the carboxylates of D21 and D69, which is similar to previously reviewed bona fide Thg1 structures, other polymerases, and BtTLP. The T arm is recognized by the opposing monomer in the MaTLP dimer; phosphate groups of U_55_ and G_57_ are involved in hydrogen-bond interactions with the protruding long arm or β-hairpin region of MaTLP [29]. Computational analysis and model structures of MaTLP–tRNA complexes have shown truncated but not full-length tRNA molecules binding to MaTLP, suggesting that TLP molecules recognize 5′-truncated tRNA molecules via the flexible β-hairpin, and terminate elongation by recognizing the acceptor stem length of its substrates [29]. 

The archaeon *I. hospitalis* encodes a minimalized TLP homolog (IhTLP) with a molecular weight of 18 kDa, which is smaller than its archaeal cousin PaTLP (25 kDa) and the bona fide Thg1 from *C. alibcans* (35 kDa). IhTLP encodes the three conserved carboxylates that Thg1 and other polymerases require to coordinate two metal ions for catalysis, but shares little overall homology to other TLP enzymes. Furthermore, the fingers domain, which are responsible for the anticodon loop and acceptor stem coordination, is significantly minimalized in IhTLP. IhTLP was found to be catalytically active in vitro, and catalyzes a significant tRNA repair reaction in vitro, adding up to 13 nucleotides to restore a truncated tRNA^His^, yet the in vivo function of IhTLP remains to be investigated [35]. While this mode of recognition is suitable for rationalizing TLP roles in repairing 5′-truncated tRNA, the prospect of alternative non-tRNA substrates for at least some members of this enzyme family raises additional questions about how this mechanism could be adapted more broadly to control 3′ to 5′ polymerase reactions. 

### 3.6. 5′-End Activation and Nucleotidyl Transfer in TLPs

Many features of the overall mechanism of 3′ to 5′ nucleotide addition appear to be shared between Thg1 and TLP members of the enzyme family. Distinct residues that participate in 5′-end activation (*ScThg1*: K44, S75, N161) and nucleotidyl transfer (*ScThg1*: R27, K96, R133) are found in similar positions in the BtTLP structure, except for the N161 residue, which is absent in TLPs. The differing effects on distinct catalytic steps of the reaction that are observed upon the mutagenesis of these residues led to the notion of partially separable active sites for 5′-end activation and nucleotidyl transfer, which appears to also be the case for TLPs [4,5,28,29,30,33]. However, several molecular details distinguish these two types of enzymes. In terms of the first 5′-end activation step of the reaction, BtTLP and the TLP from *M. xanthus* (MxTLP) can utilize GTP to activate the 5’-end of tRNA^His^ in vitro, whereas bona fide Thg1 enzymes are so far strictly ATP-dependent for catalyzing this reaction [6,23,28,40]. The crystal structures of TLP enzymes captured in the pre-activation conformation with either ATP or GTP nucleotides bound in the activation nucleotide-binding site have been determined, and confirm the roles for the residues implicated kinetically in the 5′-end activation reaction [28]. For the conserved lysine (K44 in ScThg1, K43 in BtTLP), this residue appears to be important specifically for the activation reaction with ATP, and not used for the GTP activation reaction, since the alteration of this residue in the context of the bifunctional BtTLP only affected 5′-adenylylation rates. Structural data support this ATP-specific role, since the side chain of K43 would predictably clash with the exocyclic 2′-amine of GTP bound in the active site. It is possible that ScThg1 K44 interacts with other yet-unidentified residues in the ScThg1 active site to ensure its ATP-dependent activity by preventing GTP binding in a catalytically competent conformation at this site. 

Insight into the template-dependent nucleotidyl transfer step catalyzed by TLPs was provided by the structure of MaTLP bound to a GTP analog and a 5′-truncated triphosphorylated tRNA^Phe^ (ppptRNA^Phe^Δ_1_), which mimics the activated tRNA ready for the nucleotidyl transfer. The incoming nucleotide uses Watson–Crick base-pairing and base-stacking interactions to facilitate its incorporation to the tRNA substrate [28]. In addition, binding of the incoming nucleotide seems to promote a significant structural change in the 5′-end of the tRNA substrate to accommodate the nucleotide incorporation across from the templating nucleotide. Also, no interactions between the enzyme and the base moiety of the GTP analog were observed here, supporting the idea of template-dependency exhibited by TLPs for nucleotide addition to their substrates, which is presumably distinct from the expected base-recognition capabilities for Thg1 homologs to catalyze template-independent GTP addition. In TLPs, coordination of the Mg^2+^ A to the 3′-OH of incoming NTP facilitates the nucleophilic attack on the α-phosphate of the activated tRNA substrate, which is analogous to the role of this residue in canonical polymerases. Meanwhile, the triphosphate of the incoming NTP coordinates to a third metal ion (Mg^2+^ C). It is arguable that the Mg^2+^ C seen in the active site of Thg1/TLP structures stabilizes the incoming NTP in the absence of metal coordination from other two Mg^2+^ ions (Mg^2+^ A and Mg^2+^ B), which are already coordinated to either the activating ATP that resides in the activation site or the activated tRNA substrate. Appearance of a third metal ion has also been observed in DNA pol β and DNA pol η [55,56]. Although these polymerases use the canonical two-metal ion mechanism, the third metal ion has been proposed to facilitate phosphoryl transfer to the nucleophilic 3′-OH group of the growing polynucleotide chain. Similarly, the use of a third Mg^2+^ ion (Mg^2+^ C) in TLPs seems to facilitate nucleophilic attack on the α-phosphate of the activated tRNA to promote PP_i_ release upon subsequent nucleotidyl transfer. 

## 4. Synthetic Biology Applications and Thg1/TLP Engineering

How TLP enzymes distinguish between truncated tRNA and full-length tRNA in the context of 5′-tRNA elongation or repair and how the elongation reaction is properly terminated in all cases remains to be fully solved. The proper termination of Thg1 enzymes after adding only the single G nucleotide to tRNA^His^ is intimately connected with the acquisition of the A_73_ discriminator nucleotide of tRNA^His^ in eukaryotes, as opposed to the universally conserved (and likely ancestral) C_73_ that is found in Bacteria and Archaea. Indeed, the molecular basis for the ability of the G_-1_:A_73_ base pair to trigger the termination of the addition in ScThg1 is demonstrated to result from ScThg1’s highly efficient removal of the 5′-triphosphorylated end from tRNA species that terminate in this non-Watson–Crick base pair, as described above [36]. This mechanism for termination is unlikely to be conserved among TLPs, since these enzymes strictly add Watson–Crick base pairs that are not efficient substrates for the 5′-pyrophosphatase activity [36]. The previously demonstrated capability of TLPs to catalyze extended reverse RNA polymerization [23,29,35,40] makes these proteins promising candidates for protein engineering to harness their ability to extend RNAs in the 3′ to 5′ direction in a template-dependent manner.

### 4.1. TLPs Exhibit Broader RNA Recognition Properties Than Thg1 Homologs

The ability to act on a broader range of tRNA substrates than the tRNA^His^-specific Thg1 homologs naturally positions TLP enzymes as candidates for engineering 3′ to 5′ polymerases for diverse functions. Understanding the basis for RNA recognition by various members of the Thg1 superfamily is a key element to these efforts. While recognition of the anticodon GUG is a major determinant for Thg1-catalyzed G_−1_ addition to tRNA^His^, the TLPs that have been tested to date do not depend on the presence of this sequence to be used as substrates for 3′ to 5′ polymerization [10,23]. This ability to act independently of the specific tRNA^His^ anticodon nucleotides is a necessary feature for TLPs to act outside of tRNA^His^ maturation, as has already been demonstrated by the physiological function of DdiTLP3 in mt-tRNA 5′-editing [42,44]. For this 5′-editing reaction, the ability to act on any tRNA is required, since many different tRNA species contain the genomically encoded 5′-mismatches that must be removed by 5′-editing and then repaired by the TLP prior to participation in translation [34,49,51,52,53]. Indeed, the pattern of tRNA species that have been demonstrated to undergo editing in the many diverse eukaryotes where this process has been investigated so far is quite different from organism to organism, and can involve from only a few species to nearly all of the mt-tRNA, thus suggesting that flexibility in tRNA recognition is important for this function [50]. The idea that some TLPs may act on other non-tRNA substrates also raises new questions for these enzymes and their ability to recognize various RNAs. Thus, TLP activity is not restricted to tRNA^His^ substrates, and may be engineered to further broaden their substrate specificity.

### 4.2. Steps Toward Utilizing TLPs for Targeted 3′-5′ Addition Reactions

Although eukaryotic Thg1 is mostly restricted to acting on tRNA^His^, bacterial and archaeal TLPs have been shown to exhibit broader RNA substrate specificity, suggesting that the 3′ to 5′ polymerase activity of TLPs could potentially be directed to site-specifically label diverse RNA substrates at their 5′-ends. TLPs are capable of adding all four nucleotides in a template-dependent fashion [23,29,35,40], which makes them true reverse polymerases. This could in principle also be extended to add labeled or modified nucleotides at specific positions of a given RNA substrate. This approach has previously been successful in 3′-biotinylation by the terminal nucleotidyltransferase Cid1 [38]. Other applications that we can envision include reverse polymerization on additional substrates, which would allow for applications such as the site-specific incorporation of nucleotides to the 5′-ends of tRNA transcripts that are difficult to incorporate during in vitro synthesis due to the limitations of the commonly used T7 RNA polymerase. Here, we describe several approaches that have been used successfully for this type of application.

One critical step in Thg1/TLP engineering is to broaden substrate specificity toward RNAs other than tRNA. While the tRNA^His^ GUG anticodon is not required for TLP activity, recognition of the overall tertiary tRNA structure is a conserved element for Thg1 and TLPs. Desai et al. developed a split tRNA approach, which was later successfully implemented by Nakamura et al., dividing the tRNA through the D-loop. Here, the tRNA structure is mostly provided by a guide RNA, complementary to the RNA of interest to be guanylylated [35,54]. The guided RNA approach has been successful to lead RNaseP to mRNA substrates and cleave pathogenic RNAs in cancer [57]. Thus, this approach could be used to direct Thg1 to any given RNA 5′-end when provided with a complementary sequence, resulting in a tRNA-like structure. 

An alternative approach provides an external RNA-guide template, which is used to anneal to the full-length tRNA, ideally disrupting the structure in the aminoacyl acceptor and D-stems, and thus providing a template to add the desired nucleotide(s) to the tRNA. To demonstrate the feasibility of this approach, we exploited the known inability of TLP enzymes to efficiently add a non-Watson–Crick base-paired G_−1_ to wild-type tRNA^His^ with an A_73_ discriminator nucleotide [23,47]. Then, by adding an additional RNA oligonucleotide that is complementary to the tRNA sequence, we provided an alternative template that could possibly direct the addition of a nucleotide(s) to the tRNA 5′-end in the Watson–Crick-dependent manner that is preferred by TLPs. We reasoned that if TLP enzymes are capable of accommodating an external oligonucleotide template, 3′-5′ polymerase activity could potentially be directed to add nucleotides to the 5′-end of any given RNA substrate [49].

**Figure 7:**
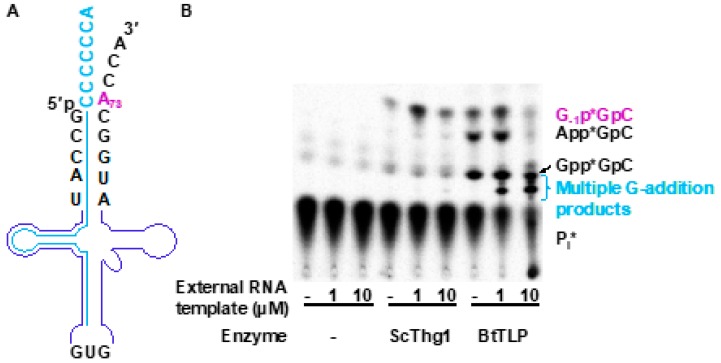
An external guide RNA oligonucleotide-mediated approach to manipulate nucleotide addition by *Bacillus thuringiensis* TLP homolog (BtTLP). (**A**) Schematic representation of tRNA^His^ (backbone in purple) hybridized to the external guide RNA oligonucleotide template (shown in blue). (**B**) Activity assay using the external guide RNA template and analyzed by thin-layer chromatography (TLC). Identities of the labeled reaction products are consistent with the known migration patterns of these species that have been validated by previous assays [23]. Nucleotide addition was tested using *Saccharomyces cerevisiae* Thg1 (ScThg1) and BtTLP with varying concentrations of the external guide RNA template (0 μM, 1 μM, and 10 μM, as indicated). In the absence of the guide RNA template, ScThg1 efficiently adds G_−1_ and in contrast, BtTLP only weakly adds G_−1_ across from A_73_ (evident from the amount of the product G_−1_p*GpC, which is labeled in purple). BtTLP instead accumulates 5′-adenylylated and 5′-guanylylated intermediates (evident from the products App*GpC and Gpp*GpC, which are labeled in black respectively). In the presence of the external guide RNA template, ScThg1 still maintains its ability to add a single G nucleotide across from A_73_ in *S. cerevisiae* tRNA^His^ (Sc-tRNA^His^, as evident from the product G_−1_p*GpC shown in purple), whereas BtTLP adds multiple G nucleotides using the 3′-CCCCCCA of the external guide RNA oligonucleotide as a template. This multiple addition is evident from the appearance of several lower migrating products, which are labeled in blue, and are absent in ScThg1-containing reactions. The precise number of G-residues added to the tRNA cannot be determined due to the inability of this TLC system to resolve the distinct species that contain more than three added G-nucleotides [9,23,44], but the pattern of migration observed here is consistent with the migration described previously for these species.

To test this, we used labeled in vitro transcribed *S. cerevisiae* tRNA^His^ (Sc-tRNA^His^) (Figure 7A). In the absence of any added oligonucleotide, ScThg1 adds G_−1_, forming a non-Watson–Crick base pair, as evident from the G_−1_p*GpC product that is observed after G-addition (Figure 7B). BtTLP only weakly adds G_−1_; however, as observed previously, it instead accumulates 5′-adenylylated and 5′-guanylylated intermediates that are the products of the first activation reaction (Figure 7B). Then, varied concentrations (1 µM and 10 µM) of an externally provided 38-mer oligonucleotide template that is complementary to 31 nucleotides on the 5′-end of Sc-tRNA^His^ were added to the assays. When this oligonucleotide anneals to Sc-tRNA^His^, it will create a 7-nucleotide 3′-overhang that can serve as a template for nucleotide addition to the 5′-end of the Sc-tRNA^His^ (Figure 7A). Here, the 3′-CCCCCCA overhang has been chosen due to the preferential G–C base pairing activity exhibited by TLP enzymes.

Interestingly, the addition of the external templating oligonucleotide did not appreciably affect the products observed in the reactions with ScThg1. Only a single G_−1_ residue was still observed, suggesting that ScThg1 is not using the multiple C-containing template that would result in multiple nucleotide addition, as previously observed for variant tRNA with this 3′-end sequence [9]. Instead, ScThg1 must be utilizing the native Sc-tRNA^His^ 3′-end instead of the oligonucleotide-bound structure as a template. This is possibly due to changes in the overall shape of tRNA^His^ when bound to the oligonucleotide, which underscores this enzyme’s stricter dependence on tRNA^His^ structure that has been demonstrated repeatedly, and also the unsuitability of Thg1 members of the 3 to 5′ polymerase family for this type of engineering approach. 

In contrast, the addition of the external templating oligonucleotide to reactions with BtTLP results in significant changes in the observed reaction products. BtTLP efficiently created lower migrating products that have previously been identified as corresponding to multiple G-nucleotide additions [31], as expected for the use of the 3′-CCCCCCA sequence as a template. This observation provides proof for the first time that TLPs are capable of accommodating an externally provided oligonucleotide, and also implies that the 3′-5′ polymerase activity of TLPs can be guided to label an RNA substrate with a template of interest. Appearance of the products corresponding to multiple G-additions in the assays containing BtTLP supports the idea that BtTLP is an enzyme with more flexibility in accommodating an unnatural template and most importantly, BtTLP is capable of changing its nucleotide addition pattern to match the template that is available. The replacement of the tRNA^His^ acceptor stem by the external oligonucleotide alone was sufficient to guide BtTLP to change its nucleotide addition preferences as opposed to Thg1. 

## 5. Conclusions

The tRNA^His^ guanylyltransferase family comprises a fascinating group of enzymes with novel catalytic activities. From the initial identification of these enzymes as the catalysts of adding a somewhat simple, albeit critical, single nucleotide to the 5′-ends of tRNA^His^ species, the mechanistic and biological complexity that is associated with the members of this family has grown significantly. New surprises about the functions of these enzymes, both engineered and on natural RNA substrates, are sure to continue to emerge, and will provide new opportunities to take advantage of 3′ to 5′ polymerization in the future.
